# The association between fibrinogen levels and severity of coronary artery disease and long-term prognosis following percutaneous coronary intervention in patients with type 2 diabetes mellitus

**DOI:** 10.3389/fendo.2023.1287855

**Published:** 2023-11-29

**Authors:** Hong Su, Yi Cao, Qiang Chen, Tao Ye, Caiyan Cui, Xu Chen, Siqi Yang, Lingyao Qi, Yu Long, Shiqiang Xiong, Lin Cai

**Affiliations:** ^1^ Department of Cardiology, The Southwest Medical University, Luzhou, Sichuan, China; ^2^ Department of Cardiology, The Third People’s Hospital of Chengdu, Affiliated Hospital of Southwest Jiaotong University, Chengdu Cardiovascular Disease Research Institute, Chengdu, Sichuan, China

**Keywords:** fibrinogen, Percutaneous coronary intervention, prognosis, The SYNTAX score, type 2 diabetes mellitus

## Abstract

**Background:**

Fibrinogen is a potential risk factor for the prognosis of CAD and is associated with the complexity of CAD. There is limited research specifically investigating the predictive role of fibrinogen in determining the severity of CAD among patients with T2DM, as well as its impact on the prognosis following PCI.

**Methods:**

The study included 675 T2DM patients who underwent PCI at the Third People’s Hospital of Chengdu between April 27, 2018, and February 5, 2021, with 540 of them remaining after exclusions. The complexity of CAD was assessed using the SYNTAX score. The primary endpoint of the study was the incidence of MACCEs.

**Results:**

After adjusting for multiple confounding factors, fibrinogen remained a significant independent risk factor for mid/high SYNTAX scores (SYNTAX score > 22, OR 1.184, 95% CI 1.022-1.373, P = 0.025). Additionally, a dose-response relationship between fibrinogen and the risk of complicated CAD was observed (SYNTAX score > 22; nonlinear P = 0.0043). The area under the receiver operating characteristic curve(AUROC) of fibrinogen for predicting mid/high SYNTAX score was 0.610 (95% CI 0.567–0.651, P = 0.0002). The high fibrinogen group (fibrinogen > 3.79 g/L) had a higher incidence of calcified lesions and an elevated trend of more multivessel disease and chronic total occlusion. A total of 116 patients (21.5%) experienced MACCEs during the median follow-up time of 18.5 months. After adjustment, multivariate Cox regression analysis confirmed that fibrinogen (HR, 1.138; 95% CI 1.010-1.284, P = 0.034) remained a significant independent risk factor for MACCEs. The AUROC of fibrinogen for predicting MACCEs was 0.609 (95% CI 0.566-0.650, P = 0.0002). Individuals with high fibrinogen levels (fibrinogen > 4.28 g/L) had a higher incidence of acute myocardial infarction (P < 0.001), MACCEs (P < 0.001), all-cause death (P < 0.001), stroke (P = 0.030), and cardiac death (P = 0.002). Kaplan-Meier analysis revealed a higher incidence of MACCEs in the high fibrinogen group (Log-Rank test: P < 0.001).

**Conclusions:**

Elevated fibrinogen levels were associated with increased coronary anatomical complexity (as quantified by the SYNTAX score) and a higher incidence of MACCEs after PCI in patients with T2DM.

## 1 Introduction

Coronary artery disease (CAD) is among the leading causes of mortality worldwide (1). Hyperglycemia, abnormal lipid metabolism, insulin resistance, and oxidative stress reactions caused by diabetes can exacerbate the development of atherosclerosis in patients (2), which negatively impacts their clinical prognosis. The SYNTAX score is commonly used to assess the complexity of coronary artery lesions and guide the selection of revascularization strategies between coronary artery bypass grafting surgery(CABG) and percutaneous coronary intervention (PCI) in patients with complex CAD (3–5). Previous studies have shown that it holds substantial predictive value in assessing the prognoses of patients undergoing percutaneous coronary intervention (6, 7). Patients can be categorized into different risk groups based on their SYNTAX scores: low risk (≤22), intermediate risk (23-32), and high risk (≥33). Higher scores indicate a greater complexity of coronary artery lesions and suggest a poorer prognosis (8, 9). The SYNTAX score derives from invasive coronary angiography, non-invasive assessments for determining the complexity of CAD might have potential benefits, as they can aid in patient stratification prior to invasive coronary angiography.

Underlying processes such as inflammation, endothelial dysfunction and enhanced coagulant activity are closely associated with the initiation and progression of atherosclerosis (10). Fibrinogen is a key component that drives blood coagulation and functions as an inflammatory factor, promoting the onset and growth of thrombosis and atherosclerosis (11, 12). Fibrinogen levels have been linked to the incidence and advancement of CAD (13–16). Moreover, it can predict the short-term and long-term risks of death and adverse cardiovascular events in patients with CAD, even those who have undergone PCI (17–22). Previous studies have established a connection between fibrinogen and cardiovascular events in patients with CAD and type 2 diabetic mellitus (T2DM) patients (23). Moreover, studies revealed that fibrinogen levels can act as an index of the severity of coronary artery lesions in patients with stable angina pectoris (SAP) (24) and acute coronary syndrome (ACS) (25). However, the ability of fibrinogen to assess the complexity of CAD in patients with T2DM remains unclear, and limited research has investigated the correlation between fibrinogen and the prognosis of patients with T2DM who undergo PCI. Therefore, the aim of this study is to investigate the association between fibrinogen levels and the complexity of CAD, as well as the prognosis after PCI in patients with T2DM.

## 2 Manuscript

### 2.1 Materials and methods

#### 2.1.1 Study design and participants

**Figure 1:**
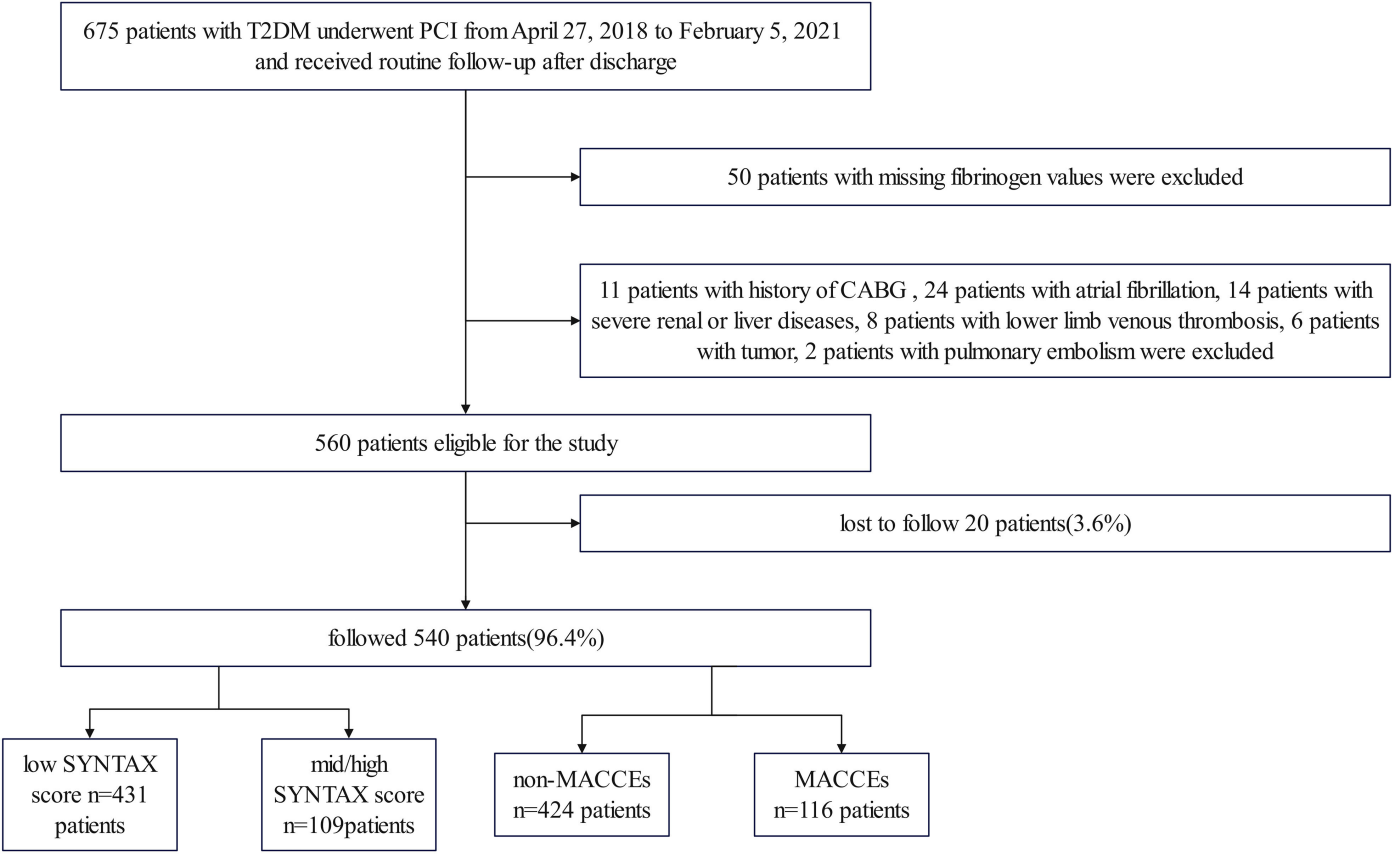
Study flowchart.

This study is a single-center, retrospective, observational cohort study. A total of 675 patients with T2DM who had undergone PCI between April 27, 2018, and February 5, 2021, at the Third People’s Hospital of Chengdu (Sichuan, China), were included in the study (**Figure 1**). After applying the inclusion and exclusion criteria, the analysis included a total of 540 patients. The cohort comprised 364 (67.4%) males and 176 (32.6%) females, with ages ranging from 27 to 97 years. The inclusion criteria encompassed individuals older than 18 years, afflicted with T2DM and CAD who had undergone PCI. Additionally, comprehensive hospitalization records, examination data, interventional surgery details, and relevant imaging data should be readily available. The exclusion criteria comprised the absence of fibrinogen levels, SYNTAX scores, or follow-up data, along with hematological, tumorous, severe liver or renal diseases, pulmonary embolism, lower limb deep vein thrombosis, atrial fibrillation, and previous coronary artery bypass grafting. The study received approval from the local ethics committee and followed the Declaration of Helsinki guidelines, including obtaining informed consent from participants.

Data on medical history, smoking status, sociodemographic information, laboratory results and procedural details of patients were extracted from their electronic medical records. The patients were followed up at 3, 6, and 12 months, and subsequently annually thereafter, via outpatient visits or phone interviews which were conducted to collect follow-up data. For patients who adhered to their prescribed outpatient clinic visits, clinical outcomes were recorded during these appointments. Any patients not present at these scheduled visits were contacted via telephone, at the corresponding time intervals, to determine whether they had experienced any serious incidents such as death, stroke, recurrent myocardial infarction, or revascularization events. In cases of patients encountering severe events — such as a recurrence of myocardial infarction or repeat revascularization — and admitted to the Third People’s Hospital of Chengdu, their pertinent data during this subsequent hospitalization were gathered to uphold the precision of the study outcomes. The median follow-up duration was recorded as 18.5 months, while the interquartile range for the follow-up period was found to be 14.4-22.6 months. The primary endpoint is major cardiovascular and cerebrovascular adverse events (MACCEs), defined as a composite of all-cause death (cardiac or non-cardiac), recurrent myocardial infarction, unplanned revascularization, and stroke. The secondary endpoints included all-cause death, cardiac death, recurrent myocardial infarction, unplanned revascularization, and stroke.

T2DM was defined according to the criteria established by the American Diabetes Association (ADA) (26). Hypertension was defined as systolic blood pressure (SBP) above 140 mmHg and/or diastolic blood pressure (DBP) above 90 mmHg or the use of antihypertensive medications (27). Medical history data included prior occurrences of hypertension, percutaneous coronary intervention, heart failure, chronic obstructive pulmonary disease, stroke, peripheral arterial disease, and chronic kidney disease. Standard biochemical techniques were employed at the Clinical Laboratory of the Third People’s Hospital of Chengdu, China to measure laboratory parameters. Residual cholesterol levels (mmol/L) were determined by subtracting the sum of High-Density Lipoprotein Cholesterol (HDL-C) and Low-Density Lipoprotein Cholesterol (LDL-C) from the total cholesterol concentration (28). The left ventricular ejection fraction (LVEF) (29) was determined using the two-dimensional modified Simpson’s method. A web-based tool named http://syntaxscore.com/ was used to compute the SYNTAX score (3). Two independent cardiologists blinded to the study protocol and baseline clinical characteristics performed this task using the procedural angiograms.

#### 2.1.2 Statistical analysis

The normality of the samples was assessed using the Shapiro-Wilk test. Due to deviation from normal distribution, continuous variables were reported as median and interquartile range. Inter-group comparisons were performed using the non-parametric rank sum test. Categorical variables were presented as frequencies and percentages. Group differences were assessed using either the Chi-square test or Fisher’s exact test.

Univariate and multivariate logistic regression analysis were adopted to determine the correlation between fibrinogen and the angiographic severity of CAD, classified as a SYNTAX score of ≤22 versus >22. Multivariate regression analyses included variables that had an unadjusted P of <0.05 after checking for collinearity. Odds ratios (ORs) with 95% confidence intervals (CIs) are used to describe the results. Restricted cubic splines (RCS) were employed to determine the potential dose-response relationship between the baseline fibrinogen level and CAD severity.

Univariate and multivariate COX survival analyses were used to identify the risk factors linked with MACCEs. Hazard ratios (ORs) with 95% confidence intervals (CIs) are used to describe the results. The Kaplan-Meier curve was constructed with end follow-up time of 1287 days used to construct survival curves and compared them through log-rank tests for time-to-event analyses of clinical endpoints.

To determine the diagnostic performance of fibrinogen in detecting the severity of CAD and MACCEs in patients with T2DM, we calculated the area under the receiver operating characteristic (ROC) curve (AUROC). The Delong test was utilized to determine the statistical significance between fibrinogen and SYNTAX score in predicting MACCEs.

All statistical analyses were conducted using SPSS version 28.0 software (IBM Corporation, New York, NY, USA), MedCalc 20.100 and R version 4.2.3 software (R Foundation for Statistical Computing, Vienna, Austria). A P < 0.05 was considered statistically significant.

### 2.2 Results

#### 2.2.1 Baseline characteristics between low (SYNTAX score ≤ 22) and mid/high risk (SYNTAX score > 22) groups

The baseline characteristics of 540 patients based on of the SYNTAX score are shown in **Table 1**. Compared to patients in the low SYNTAX score group, though in the mid/high SYNTAX score group had higher levels of heart rate, troponin-T, B-type natriuretic peptide, cystatin C, cholesterol, residual cholesterol, apolipoprotein B, homocysteine, fibrinogen, and D-dimer, and lower levels of albumin, direct bilirubin, indirect bilirubin, red blood cell count, hemoglobin, and LVEF. Patients with mid/high SYNTAX score also had a larger number and length of stents. Furthermore, these patients had higher prevalence rates of chronic kidney disease, acute myocardial infarction (AMI), left main lesion, multivessel disease (MVD), calcified lesions, thrombosis, long lesion, and chronic total occlusion (CTO).

**Table 1:** Baseline characteristics between low (SYNTAX score ≤ 22) and mid/high risk (SYNTAX score > 22) groups [Median (IQR)].

Variables	Syntax scores ≤ 22 (n=431)	Syntax scores > 22(n=109)	P
Male, n (%)	290(67.3)	73(67.0)	0.950
Age, years	69(62,76)	69(61,78)	0.653
BMI, kg/m^2^	24.52(22.76,26.82)	24.22(22.74,26.38)	0.514
Hypertension, n (%)	324(75.2)	77(70.6)	0.334
Previous PCI, n (%)	47(10.9)	7(6.4)	0.163
Previous heart failure, n (%)	19(4.4)	7(6.4)	0.380
COPD, n (%)	13(3.0)	2(1.8)	0.731
Previous stroke, n (%)	27(6.3)	5(4.6)	0.508
Peripheral arterial disease, n (%)	7(1.6)	0	0.180
Chronic kidney disease, n (%)	16(3.7)	9(8.3)	0.044
Smoking, n (%)	200(46.5)	45(40.9)	0.950
SBP, mmHg	133(119.75,147.00)	132(117.75,150.00)	0.705
DBP, mmHg	77(67.00,85.00)	77(69.75,87.00)	0.243
Heart rate, bpm	77.50(69.00,88.25)	80(71.00,90.00)	0.017
Troponin-T, pg/ml	29.12(12.16,402.88)	137.50(18.06,1256.00)	<0.001
BNP, pg/ml	97.40(40.70,291.00)	210.10(53.20,724.95)	<0.001
Creatinine, umol/l	76.80(64.00,95.70)	80.75(64.63,108.40)	0.117
Uric acid, umol/l	367.10(295.35,439.85)	372.00(302.00,450.10)	0.542
Cystatin c, mg/l	1.18(0.98,1.47)	1.30(1.04,1.71)	0.019
FBG, mmol/l	7.90(6.11,10.60)	7.91(6.15,10.88)	0.531
HbA1c, mmol/l	7.50(6.70,8.70)	7.70(6.70,9.00)	0.546
Triglycerides, mmol/l	1.60(1.13,2.30)	1.70(1.16,2.79)	0.345
Cholesterol, mmol/l	4,18(3.46,4.97)	4.31(3.69,5.59)	0.047
HDL-C, mmol/l	1.10(0.91,1.27)	1.11(0.94,1.31)	0.269
LDL-C, mmol/l	2.53(1.95,3.13)	2.65(2.11,3.46)	0.231
Residual cholesterol, mmol/l	0.52(0.35,0.75)	0.62(0.43,1.31)	<0.001
Lipoprotein(a), mg/l	94.30(48.10,265.80)	112.40(51.95,265.50)	0.577
Apo(A), mmol/l	1.21(1.04,1.37)	1.18(1.02,1.42)	0.834
Apo(B), mmol/l	0.80(0.61,1.01)	0.87(0.70,1.12)	0.027
Homocysteine, umol/l	13.30(10.50,17.85)	14.25(10.68,18.98)	0.318
Albumin, g/l	40.00(37.10,42.52)	37.45(35.10,40.93)	<0.001
AST, IU/l	25.05(18.23,42.33)	24.45(17.53,42.23)	0.739
ALT, IU/l	25.70(17.90,40.60)	23.75(15.25,41.98)	0.191
Direct bilirubin, umol/l	2.69(2.04,3.78)	2.37(1.75,3.26)	0.021
Indirect bilirubin, umol/l	9.92(7.48,13.24)	8.91(6.77,11.71)	0.030
Fibrinogen, g/l	3.52(3.00,4.34)	3.99(3.32,4.72)	<0.001
D-dimer, mg/l	0.34(0.20,0.65)	0.50(0.28,0.93)	<0.001
PT, s	13.70(12.50,16.50)	14.20(12.50,16.85)	0.421
INR	0.95(0.91,1.01)	0.95(1.91,1.01)	0.892
APTT, s	37.90(34.93,41.10)	36.45(33.93,40.33)	0.112
RBC count, *10^12/l	4.37(3.97,4.75)	4.30(3.63,4.58)	0.009
Hemoglobin, g/l	135.00(123.00,147.00)	127.00(108.75,140.75)	<0.001
WBC count, *10^9/l	6.98(5.72,8.89)	7.11(5.92,9.27)	0.396
Platelet count, *10^9/l	161.00(131.00,200.00)	173.50(138.75,207.00)	0.067
LVEF, n (%)	58.00(53.00,62.00)	55.00(43.00,60.00)	<0.001
AMI, n (%)	174(40.4)	65(59.6)	<0.001
Diagnosis, n%			<0.001
SAP	69(16.0)	10(9.2)	
UA	188(43.6)	34(31.2)	
NSTEMI	79(18.3)	38(34.9)	
STEMI	95(22.0)	27(24.8)	
Angiographic data			
LM, n (%)	10(2.3)	14(12.8)	<0.001
MVD, n (%)	298(69.1)	99(90.8)	<0.001
Calcified lesions, n (%)	54(12.7)	41(39.4)	<0.001
Thrombosis, n (%)	39(9.2)	11(10.6)	0.667
Long lesion, n (%)	86(20.0)	36(33.0)	0.004
CTO, n (%)	67(15.8)	47(45.2)	<0.001
Number of stents	1(1,2)	2(1,3)	<0.001
Length of stents, mm	32.00(20.00,49.50)	52.00(33.00,79.00)	<0.001

Data are presented as median (IQR) or n (%). BMI, body mass index; PCI, percutaneous coronary intervention; COPD, chronic obstructive pulmonary disease; SBP, systolic blood pressure; DBP, diastolic blood pressure; BNP, B-type natriuretic peptide; FBG, fasting blood glucose; HbA1C, glycosylated hemoglobin A1c; HDL-C, High-Density Lipoprotein Cholesterol, LDL-C, Low-Density Lipoprotein Cholesterol; Apo(A), Apolipoprotein A; Apo(B), Apolipoprotein B; AST, aspartate transaminase; ALT, alanine transaminase; PT, prothrombin time; INR, international normalized ratio; APTT, activated partial thromboplastin time; RBC count, red blood cell count; WBC count, white blood cell count; LVEF, left ventricular ejection fraction; AMI, acute myocardial infarction; SAP, stable angina pectoris, UA, unstable angina; NSTEMI, non-ST-segment elevation myocardial infarction; STEMI, ST-segment elevation myocardial infarction; LM, left main disease; MVD, multivessel disease; CTO, chronic total occlusion.

#### 2.2.2 The correlation between fibrinogen and severity of CAD inT2DM patients

Univariate and multivariate logistic regression analysis of the association between the multiple characteristics and mid/high SYNTAX score are shown in **Table 2**. As noted in **Table 2**, the univariate logistic regression analysis indicated that heart rate, troponin-T, B-type natriuretic peptide, cholesterol, residual cholesterol, albumin, indirect bilirubin, fibrinogen, hemoglobin and LVEF were identified as potential risk factors for having a mid/high SYNTAX score (SYNTAX score > 22). Multivariate analysis was performed to assess the significant predictors identified through univariate screening (univariate P < 0.05). Cholesterol, as a component of the residual cholesterol, was not included in the multivariable logistic regression model in order to avoid any potential interactions. Furthermore, chronic kidney disease (OR, 2.329; 95%CI 1.000-5.433, p = 0.050) also was included in the multivariable logistic regression model. After checking for collinearity, the multivariate logistic regression analysis revealed that fibrinogen was an independent predictor of a mid/high SYNTAX score (SYNTAX score > 22, OR,1.184; 95% CI 1.022-1.373, P = 0.025).

**Table 2:** Univariate and multivariate logistic regression analysis of syntax.

Variables	Univariate analysis		Multivariate analysis	
	OR (95%CI)	P	OR (95%CI)	P
Chronic kidney disease	2.329(1.000-5.433)	0.050	1.049(0.379-2.899)	0.927
Heart rate	1.0180(1.004-1.033)	0.012	1.010(0.994-1.027)	0.214
Troponin-T	1.000(1.000-1.000)	0.031	1.000(1.000-1.000)	0.952
BNP	1.001 (1.000-1.001)	0.029	1.001(1.000-1.001)	<0.001
Cystatin c	0.994(0.957-1.031)	0.734		
Cholesterol	1.262(1.079-1.477)	0.004	/	/
Residual cholesterol	2.254(1.417-3.584)	<0.001	2.293(1.423-3.696)	<0.001
Apo(B)	1.026(0.871-1.209)	0.759		
Albumin	0.946(0.906-0.988)	0.027	1.006(0.956-1.006)	0.121
Direct bilirubin	0.886(0.776-1.010)	0.071		
Indirect bilirubin	0.947(0.903-0.993)	0.034	0.954(0.905-1.006)	0.080
Fibrinogen	1.167(1.020-1.335)	0.025	1.184(1.022-1.373)	0.025
D-dimer	0.994(0.962-1.027)	0.709		
RBC count	1.009(0.985-1.033)	0.477		
Hemoglobin	0.986(0.977-0.994)	0.001	0.990(0.978-1.002)	0.105
LVEF	0.963(0.943-0.997)	<0.001	0.958(0.936-0.981)	<0.001

Data are presented as OR (95%CI). Abbreviations as shown in **Table 1**.

**Figure 2:**
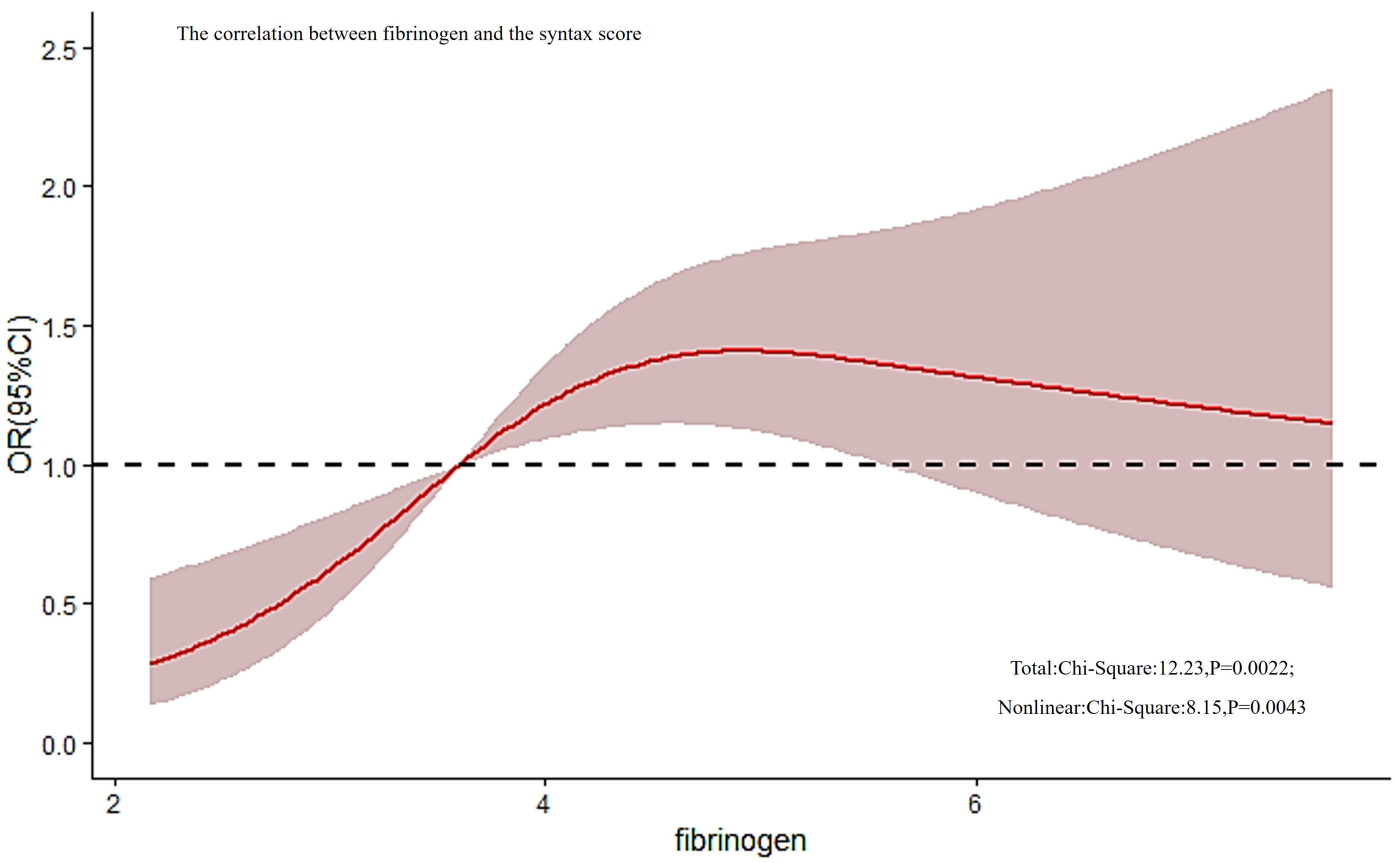
The correlation between fibrinogen and the SYNTAX score. RCS analysis found that there was a non-linear correlation between fibrinogen and SYNTAX score (overall model validity: total: X^2 = ^12.23, P = 0.0022, nonlinear: X^2 = ^8.15, P = 0.0043). RCS, restricted cubic spline; SYNTAX, score Synergy Between Percutaneous Coronary Intervention score.

The spearman’s correlation analysis revealed an extremely weak positive correlation between fibrinogen and SYNTAX scores (r = 0.109, P = 0.011). However, the RCS results indicated that there was a potential dose-response relationship between fibrinogen and the risk of a mid/high SYNTAX score, as shown in **Figure 2**. Further testing uncovered a non-linear correlation between fibrinogen and SYNTAX score (overall model validity: total: X^2 = ^12.23, P = 0.0022, nonlinear: X^2 = ^8.15, P = 0.0043).

**Figure 3:**
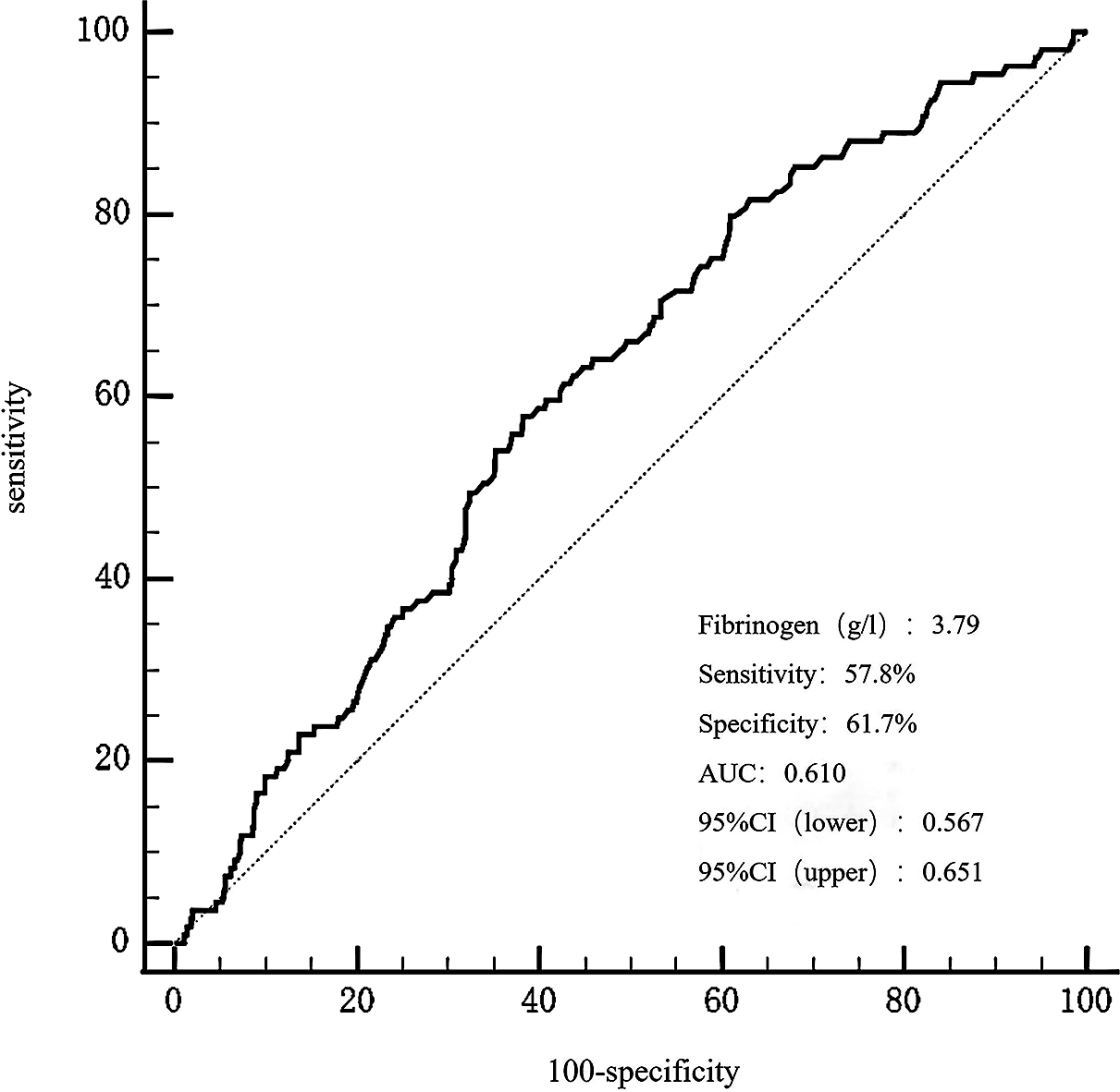
The ROC curves for predicting a mid/high SYNTAX score by fibrinogen. The area under the ROC curve of the fibrinogen for predicting a mid/high SYNTAX score (> 22) was 0.610 (95% CI 0.567–0.651, P = 0.0002), respectively. ROC, curve receiver operating characteristic curve; SYNTAX, score Synergy Between Percutaneous Coronary Intervention score.

The receiver operating characteristic (ROC) curve analysis exhibited that fibrinogen predicted the mid/high SYNTAX score, with an area under the curve (AUC) of 0.610, 95% confidence interval (CI) 0.567-0.651, and p = 0.0002 (**Figure 3**). Fibrinogen’s optimal cut-off value for predicting the mid/high SYNTAX score, with a maximum sensitivity of 57.8% and specificity of 61.7%, was 3.79g/L. This cut-off value divided patients into two groups: those with fibrinogen levels ≤ 3.79g/L and those with levels > 3.79g/L (**Supplementary Material Table S1**). Patients with fibrinogen levels > 3.79g/L exhibited significantly greater prevalence of previous stroke and chronic kidney disease when compared to those with fibrinogen levels ≤ 3.79g/L. Moreover, the fibrinogen > 3.79g/L group showed significantly higher levels of heart rate, troponin-T, B-type natriuretic peptide, creatinine, cystatin C, fasting blood glucose, lipoprotein(a), apolipoprotein B, D-dimer, white blood cell count, platelet count, the SYNTAX score, number of stents, and stent length, but significantly lower levels of albumin, RBC count, hemoglobin, and LVEF. Additionally, patients in the higher fibrinogen group had more frequent cases of AMI, calcified lesions, and MACCEs.

#### 2.2.3 Baseline characteristics between MACCEs and non-MACCEs

During the median follow-up time 18.5 months, 116 (21.5%) out of 540 patients reached a clinical endpoint - including all-cause deaths in 35 patients (6.5%), recurrent myocardial infarction in 15 patients (2.8%), unplanned revascularization in 60 patients (11.1%), and stroke in 23 patients (4.3%). Furthermore, 17 of the patients demonstrated several endpoints as classified in the MACCEs. The baseline characteristics of the patients are provided in **Table 3**. Among the MACCEs group, there was a higher occurrence of chronic kidney disease, MVD, calcified lesions, and CTO. The MACCEs group also exhibited elevated levels of heart rate, creatinine, uric acid, cystatin C, lipoprotein (a), homocysteine, fibrinogen, and D-dimer, in addition to a high SYNTAX score. In contrast, the MACCEs group had a low level of alanine aminotransferase, aspartate aminotransferase and LVEF (P < 0.05).

**Table 3:** Baseline characteristics between MACCEs and non-MACCEs [Median (IQR)].

Variables	Non-MACCEs(n=424)	MACCEs(n=116)	P
Male, n (%)	288(67.9)	76(65.5)	0.624
Age, years	69(61,76)	69(61.25,78)	0.653
Hypertension, n (%)	313(75.8)	88(75.9)	0.656
Previous PCI, n (%)	41(9.7)	13(11.2)	0.625
Previous heart failure, n (%)	17(4.0)	9(7.8)	0.095
COPD, n (%)	12(2.8)	3(2.6)	0.887
Previous stroke, n (%)	29(6.8)	3(2.6)	0.086
Peripheral arterial disease, n (%)	7(1.7)	0	0.164
Chronic kidney disease, n (%)	15(3.5)	10(8.7)	0.020
Smoking, n (%)	190(44.5)	55(46.6)	0.791
SBP, mmHg	132(119,148)	138(120,149.25)	0.168
DBP, mmHg	77(67.50,85)	76(68.75,84)	0.882
Heart rate, bpm	78(67.75,87)	80(72,90)	0.010
Troponin-T, pg/ml	29.13(12.11,458.30)	48.91(16.47,402.88)	0.217
BNP, pg/ml	100.85(42.05,348.73)	151.35(48.68,656.20)	0.072
Creatinine, umol/l	76.60(63.70,94.80)	82.20(69.00,116.10)	0.006
Uric acid, umol/l	362.40(293.45,435.15)	392.40(313.58,469.48)	0.014
Cystatin c, mg/l	1.16(0.98,1.44)	1.34(1.05,1.82)	<0.001
FBG, mmol/l	7.96(6.12,10.99)	7.51(6.05,10.20)	0.231
HbA1c, mmol/l	7.60(6.70,8.70)	7.50(6.60,9.30)	0.586
Triglycerides, mmol/l	1.61(1.12,2.39)	1.58(1.19,2.40)	0.792
Cholesterol, mmol/l	4.26(3.45,5.16)	4.12(3.53,5.03)	0.784
HDL-C, mmol/l	1.10(0.92,1.28)	1.09(0.93,1.28)	0.934
LDL-C, mmol/l	2.56(1.95,3.21)	2.49(2.03,3.11)	0.618
Residual cholesterol, mmol/l	0.55(0.36,0.78)	0.56(0.40,0.79)	0.712
Lipoprotein(a), mg/l	89.95(45.73,264.03)	132.15(63.90,299.45)	0.022
Apo(A), mmol/l	1.22(1.04,1.37)	1.19(1.03,1.41)	0.973
Apo(B), mmol/l	0.82(0.62,1.04)	0.82(0.67,1.03)	0.955
Homocysteine, umol/l	13.00(10.30,17.60)	15.10(11.25,20.73)	0.005
Albumin, g/l	39.80(36.80,42.43)	39.05(36.00,41.30)	0.085
AST, IU/l	25.40(18.40,44.85)	22.45(16.60,31.33)	0.014
ALT, IU/l	25.70(17.90,41.98)	21.30(15.40,35.60)	0.018
Direct bilirubin, umol/l	2.55(1.92,3.77)	2.78(2.06,3.50)	0.605
Indirect bilirubin, umol/l	9.85(7.39,12.86)	9.20(7.39,11.75)	0.285
Fibrinogen, g/l	3.52(3.01,4.27)	3.94(3.28,4.97)	<0.001
D-dimer, mg/l	0.41(0.26,0.77)	0.46(0.30,0.97)	0.041
PT, s	13.80(12.50,16.70)	13.60(12.52,16.30)	0.890
INR	0.95(0.91,1.00)	0.95(0.92,1.01)	0.332
APTT, s	37.60(34.80,40.75)	37.95(34.73,42.28)	0.550
RBC count, *10^12/l	4.37(3.97,4.70)	4.31(3.72,4.68)	0.170
Hemoglobin, g/l	133.00(121.50,146.00)	131.50(111.75,143.25)	0.085
WBC count, *10^9/l	6.97(5.64,9.01)	7.15(5.86,8.92)	0.526
Platelet count, *10^9/l	163.00(131.00,204.00)	170.00(135.00,204.00)	0.463
LVEF, %	58(52,62)	56(45,61)	0.006
Syntax score	14(8,20)	19(12,27)	<0.001
AMI, n%	186(43.9)	53(45.7)	0.726
Diagnosis, n%			0.854
SAP	64(15.1)	15(12.9)	
UA	174(41.0)	48(41.4)	
NSTEMI	89(21.0)	28(24.1)	
STEMI	97(22.9)	25(21.6)	
Angiographic data			
LM, n (%)	16(3.8)	8(6.9)	0.148
MVD, n (%)	294(69.3)	103(88.8)	<0.001
Calcified lesions, n (%)	64(15.1)	31(29.5)	<0.001
Thrombosis, n (%)	41(9.7)	9(8.6)	0.725
Long lesion, n (%)	95(22.4)	27(23.3)	0.843
CTO, n (%)	79(18.7)	35(33.3)	<0.001
Number of stents	1(1,2)	1(1,2)	0.117
Length of stents, mm	33.00(20.00,56.00)	33(23.00,56.00)	0.261

Data are presented as median (IQR) or n (%). Abbreviations as shown in **Table 1**.

#### 2.2.4 The correlation between fibrinogen and MACCEs

Univariate Cox regression analysis was conducted on numerous variables, including chronic kidney disease, heart rate, creatinine, uric acid, cystatin C, lipoprotein (a), homocysteine, AST, ALT, fibrinogen, D-dimer, LVEF, the SYNTAX score, MVD, calcified lesions, and CTO (P < 0.05). Age and female gender were included in the analysis as potential risk factors for MACCEs. The results are shown in **Table 4**. Based on the outcomes of the univariate Cox regression analysis, multiple factors, including chronic kidney disease, creatinine, uric acid, alanine aminotransferase, aspartate aminotransferase, fibrinogen, LVEF, the SYNTAX score, MVD, calcified lesions, and CTO, were included in the multivariate model (P < 0.05). Following adjustment for various confounding variables, fibrinogen (HR, 1.138; 95% CI 1.010-1.284, P= 0.034) was established as an independent risk factor for MACCEs.

**Table 4:** Univariate and multivariate COX regression analysis of MACCEs.

Variables	Univariate analysis		Multivariate analysis	
	HR (95%CI)	P	HR (95%CI)	P
Chronic kidney disease	2.519(1.163-5.457)	0.019	1.202(0.388-3.724)	0.750
Age	1.000(0.982-1.019)	0.974		
Female	1.031(0.687-1.549)	0.882		
Heart rate	1.017(0.997-1.037)	0.632		
Creatinine	1.001(1.000-1.002)	0.013	1.000(0.999-1.002)	0.511
Uric acid	1.002(1.001-1.004)	0.006	1.002(1.000-1.004)	0.020
Cystatin c	1.008(0.992-1.024)	0.340		
Lipoprotein(a)	1.001(1.000-1.001)	0.069		
Homocysteine	1.013(0.997-1.029)	0.101		
AST	1.000(1.000-1.001)	0.037	0.999(0.998-1.002)	0.456
ALT	1.000(1.000-1.001)	0.030	0.999(0.997-1.002)	0.609
Fibrinogen	1.147(1.033–1.274)	0.010	1.138(1.010-1.284)	0.034
D-dimer	1.031(0.945-1.123)	0.495		
LVEF	0.976(0.959-0.994)	0.009	0.998(0.976-1.019)	0.834
Syntax score	1.073(1.048-1.099)	<0.001	1.051(1.023-1.081)	<0.001
MVD	2.938(1.608-5.366)	<0.001	1.874(0.995-3.530)	0.052
Calcified lesions	2.411(1.571-3.698)	<0.001	1.887(1.177-3.025)	0.008
CTO	1.891(1.253-2.8522)	0.002	1.283(0.800-2.057)	0.301

Data are presented as HR (95%CI). Abbreviations as shown in **Table 1**.

**Figure 4:**
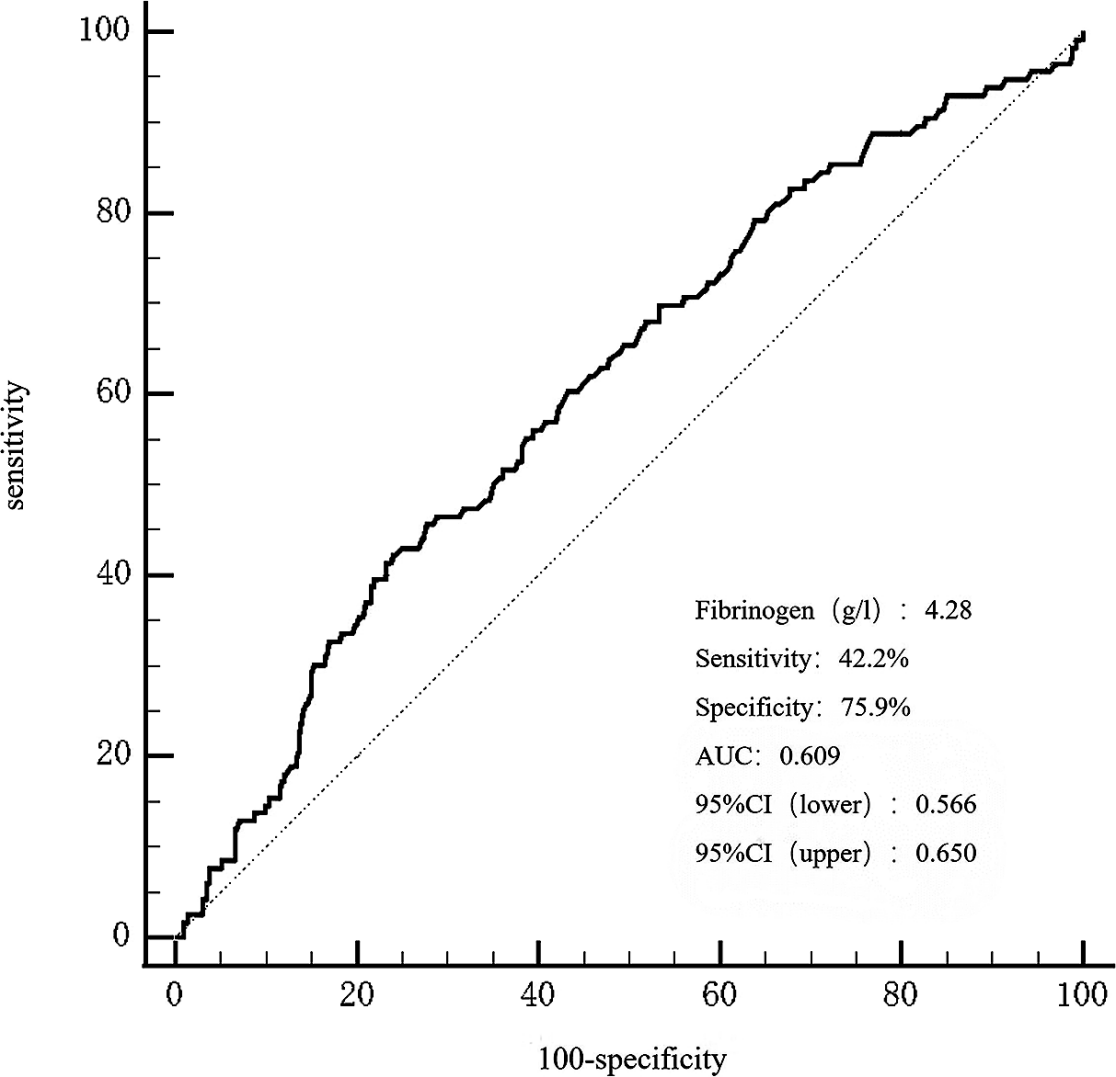
The ROC curves for predicting MACCEs. The area under the ROC curve of the fibrinogen for predicting MACCEs was 0.609 (95% CI 0.566–0.650, P < 0.001), respectively. ROC, curve receiver operating characteristic curve; MACCEs, major adverse cardiovascular and cerebrovascular events.

The area under the receiver operating characteristic curve(AUROC) of fibrinogen in predicting MACCEs was 0.609 (95% CI 0.566-0.650, P = 0.0002), as presented in **Figure 4**. The optimal cut-off value for predicting MACCEs with maximal sensitivity (42.24%) and specificity (75.9%) was 4.28g/L. Patients were divided into two groups based on the optimal cutoff value of fibrinogen to predict MACCEs: low fibrinogen levels ≤ 4.28g/L and high fibrinogen levels > 4.28g/L (**Supplementary Material Table S2**). A greater occurrence of patients with a history of stroke and chronic kidney disease was observed in the high fibrinogen group. Comparatively, patients with high fibrinogen levels recorded increased levels of heart rate, troponin T, B-type natriuretic peptide, creatinine, cystatin C, fasting blood glucose, lipoprotein (a), D-dimer, international normalized ratio, activated partial thromboplastin time, white blood cell count, platelet count, the SYNTAX score, number of stents, and stent length. In contrast, this group had lower levels of apolipoprotein A, albumin, red blood cell count, hemoglobin, and LVEF.

**Figure 5:**
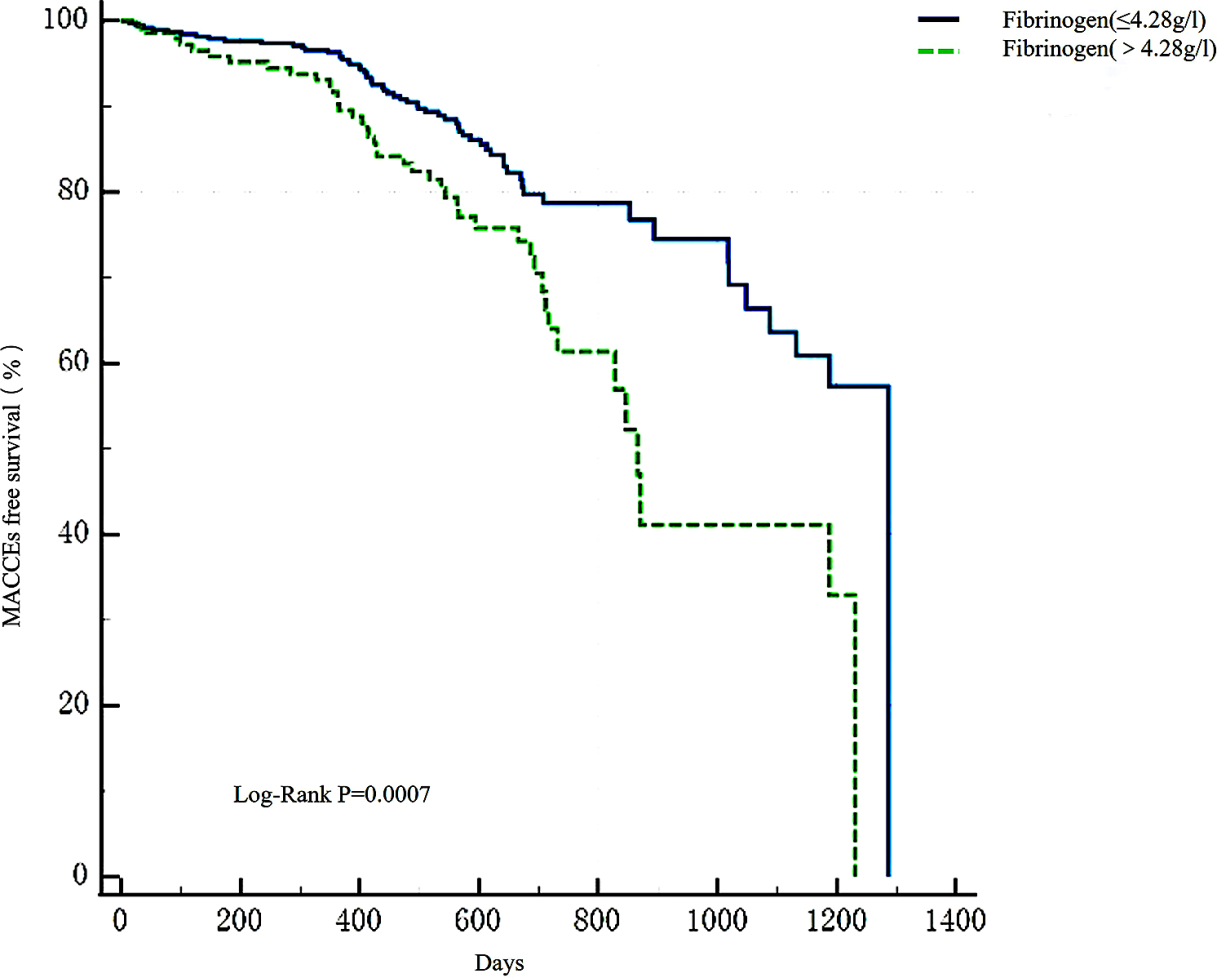
Kaplan-Meier survival analysis of MACCEs. Kaplan-Meier survival analysis found that there was a higher occurrence of MACCEs in the high-fibrinogen group (Log-Rank test: P = 0.0002). MACCEs, main adverse cardiovascular and cerebrovascular events.

Apparently, the higher fibrinogen group had a higher incidence of AMI and MACCEs (P < 0.001) (**Supplementary Material Table S2**). Patients with high fibrinogen levels had a significantly greater incidence of all-cause death (P < 0.001) and stroke (P = 0.030). Moreover, this group had a higher probability of cardiac death (P = 0.002). However, there was no apparent difference in unplanned revascularization and recurrent myocardial infarction occurrence between the two groups during the follow-up (P > 0.05). As illustrated in Kaplan-Meier analysis (**Figure 5**), the high-fibrinogen group recorded a much higher MACCEs rate (Log-Rank test: P < 0.001).

#### 2.2.5 Subgroup analysis

Subgroup analysis showed that fibrinogen was independently associated with mid/high SYNTAX risk and MACCEs in patients with ACS (**Supplementary Material Tables S3-S4**). In the total patient study, fibrinogen was an independent predictor of MACCEs, all-cause death, and cardiac death in patients with diabetes and CAD after PCI. Further analysis in subgroup showed that the predictive value of fibrinogen for poor prognosis in ACS patients mainly came from patients with unstable angina pectoris. The results showed that after adjustment, fibrinogen was an independent predictor of MACCEs, all-cause death, and repeat revascularization in patients with unstable angina pectoris (**Supplementary Material Table S4**), and fibrinogen can predict the risk of mid/high SYNTAX in UA patients, as well as the risk of MACCEs, all-cause death, and cardiac death (**Supplementary Material Table S5**).

### 2.3 Discussion

The present study demonstrates that elevated fibrinogen levels are independently associated with higher coronary anatomical complexity (assessed using a SYNTAX score > 22) and an increased risk of major adverse cardiac and cerebrovascular events in patients with T2DM who undergo PCI.

Clinical guidelines recommend the utilization of the SYNTAX score as a reliable tool for evaluating the complexity of CAD in patients with multi-vessel lesions (30). Previous studies have indicated the predictive capability of certain biomarkers, such as the Triglyceride glucose index (31) and C-reactive protein (32), in relation to the SYNTAX score.

The pathogenesis of coronary atherosclerosis involves various local inflammatory mechanisms such as endothelial dysfunction, leukocyte migration, extracellular matrix degradation, and platelet activation (10). Fibrinogen, primarily synthesized by the liver, not only serves as a crucial factor in blood coagulation but also as an acute inflammatory mediator (33). It is observed that elevated fibrinogen levels are present in various chronic inflammatory conditions, including diabetes and atherosclerosis (34, 35). Fibrinogen plays an essential role in all stages of atherosclerosis development, from initial leukocyte recruitment to eventual formation of atherosclerotic plaques (36), leading to CAD or adverse cardiovascular events through various mechanisms, including increasing plasma viscosity, inducing reversible red blood cell aggregation, binding with receptors on platelet membranes to induce platelet aggregation, and the formation of fibrin and fibrin degradation products that stimulate smooth muscle cell proliferation and migration (37). Elevated fibrinogen levels may be due to acute complications of vascular disease caused by serious events, including acute thrombosis and enhanced clotting activity resulting from impaired fibrinolytic function. In our study, high fibrinogen level group had a higher incidence of calcified lesions and an elevated trend of more multivessel disease and chronic total occlusion. It follows that high fibrinogen level may be a manifestation of more complex coronary atherosclerotic lesions. In addition, our findings showed an extremely weak positive correlation that was statistically significant (Spearman’s correlation analysis: r = 0.109, P = 0.011) and a non-linear correlation between fibrinogen and the SYNTAX scores (X^2 = ^8.15, and P = 0.0043). It confirmed that fibrinogen can predict the risk of mid/high SYNTAX scores in patients with type 2 diabetes (OR, 1.184; 95% CI 1.022-1.373, P = 0.025). This point is supported by previous researches. Kurtul A et al. (24) showed plasma fibrinogen to be an independent predictor of intermediate-high syntax scores (OR 1.008, 95% CI: 1.005-1.010, p < 0.001). The ROC curve analysis showed that fibrinogen predicted the intermediate-high syntax scores of patients with diagnosis of ACS with an area under the curve of 0.812 (95% CI: 0.778-0.846). Tabakcı MM et al. (25) observed a positive linear correlation between plasma fibrinogen levels and the syntax score (Spearman’s correlation analysis: r = 0.535, P <.001). They confirmed that fibrinogen could predict the complexity of coronary lesions in patients with stable angina pectoris (AUROC: 0.72, 95% CI 0.61-0.82, P < 0.001), even though merely 134 patients with stable angina pectoris were included. Contrarily, our subgroup analysis yielded a disparate result that might stem from differing study populations. In addition, while our study enlisted patients afflicted with both T2DM and CAD, it comprised only 79 patients with stable angina. Such a limited participant size might have restricted sample representativeness. In the context of ACS patients, fibrinogen’s capability to forecast a mid/high SYNTAX score was predominantly observed in patients with unstable angina pectoris, rather than in patients suffering from myocardial infarction(**Supplementary Material Tables S3, S5**). This can be attributed to the significant correlation found between fibrinogen and the SYNTAX score in patients with ACS(P=0.005) and unstable angina pectoris(P=0.031), and this correlation was not significant in patients with myocardial infarction(P=0.071).

Earlier studies have established an association between fibrinogen levels and the occurrence (38) or adverse clinical outcomes of CAD, encompassing both stable CAD (39, 40) and ACS (22). Additionally, increased fibrinogen levels have been associated with adverse clinical results in patients undergoing percutaneous coronary intervention (PCI) (18, 22, 41). Despite Ferraro S et al. discovering no discernible correlation between fibrinogen levels and adverse clinical outcomes post-PCI in patients with ST-segment elevation myocardial infarction (STEMI) (42), Wasilewski J et al. (43) have demonstrated that high baseline fibrinogen concentration is an independently risk factor of no tissue reperfusion in STEMI treated with successful primary PCI. This phenomenon could potentially be attributed to changes in rheology caused by increased fibrinogen, including increased blood viscosity and enhanced platelet aggregation leading to heightened microcirculatory resistance.

In patients with stable CAD, fibrinogen has been independently associated with an increased occurrence of cardiovascular events in those with T2DM (23). The absence of this phenomenon in our study could potentially be attributed to an insufficient sample size(79 patients vs 1422 patients). Another notable observational study has reported that fibrinogen is associated with an increased risk of MACCEs, especially in patients with diabetes and prediabetes (39). In terms of patients who underwent PCI, Zhang L et al. (22) demonstrated a positive association between elevated fibrinogen levels and the occurrence of MACCEs in patients with ACS, particularly those with diabetes mellitus. Another large cohort study in CAD patients after PCI showed similar results (44), assessing long-term mortality rates for both all-cause and cardiac events. In this study, we observed a significant association between elevated fibrinogen levels and a high risk of MACCEs following PCI in patients with T2DM at a median follow-up time of 18.5 months, particularly a high risk of all-cause mortality. Stroke incidence was higher in the elevated fibrinogen group compared to the low fibrinogen group (P = 0.03); however, this difference may be due to baseline distinctions in stroke events (P = 0.04). Though there was a tendency towards an increase in unplanned revascularization and recurrent myocardial infarction in the elevated fibrinogen group, no statistically significant difference was observed in the incidence of unplanned revascularization (P = 0.318) or recurrent myocardial infarction (P = 0.102) between the two groups (**Supplementary Material Table S1, Figure F1**). Concurrently, the subgroup analysis indicated that fibrinogen’s predictive capability for adverse prognosis predominantly originated from patients diagnosed with unstable angina pectoris (**Supplementary Material Tables S4, S5**). However, it bore no significant relation to adverse events like MACCEs found in patients with myocardial infarction. These findings are consistent with a previous large single-center study that focused on CAD patients who receive PCI (41) and another research study involving STEMI patients (42). However, the ADVANCE study, a case-cohort investigation involving 3,865 patients with T2DM and cardiovascular diseases or risk factors, indicated that only interleukin-6 presented a statistical significance in predicting macrovascular events and mortality following adjustment for IL-6, CRP, and fibrinogen (45). This difference may be attributed to the inclusion of patients with T2DM and one or more additional cardiovascular risk factors instead of T2DM and cardiovascular diseases. Moreover, Chinese and Indian subjects were excluded from the ADVANCE study, which could be another reason for the results disparity.

Studies have demonstrated the benefits of anti-inflammatory therapy in both chronic coronary disease (46) and ACS (47). In addition, in the ECAT Angina Pectoris Study (48), a prospective study for investigating the associations between base-line level of hemostatic factors and coronary events, coronary events were associated with higher fibrinogen levels, and in the case of high cholesterol levels, the risk of coronary events was still low as long as the fibrinogen level was controlled at a low level. The result is consistent with a pathogenetic role of impaired fibrinolysis, endothelial-cell injury, and inflammatory activity in the progression of coronary artery disease. Therefore, the role of fibrinogen in the pathogenesis of CAD and the promotion of atherosclerosis should be taken into account. Fibrinogen has been linked to mid/high SYNTAX risk and increased MACCEs risk among patients with T2DM after PCI. However, fibrinogen’s predictive ability for MACCEs risk is not entirely dependent on the SYNTAX score’s predictive capability. Firstly, we conducted a comparison between fibrinogen and the SYNTAX score to assess their ability in predicting MACCEs within this study population. The Delong test revealed no statistically significant difference (Z=1.162, P=0.245) in the predictive capacity of MACCEs between fibrinogen and the SYNTAX score. It suggested that fibrinogen possesses similar predictive power to the SYNTAX score in forecasting MACCEs among patients with T2DM. And in our study, no significant difference was observed in residual SYNTAX score levels among patients with T2DM after revascularization, regardless of the fibrinogen-level groups (3.79g/l or 4.28g/l) (**Supplementary Material Tables S1, S2**) (P > 0.05). But the results demonstrated a higher incidence of MACCEs in the high fibrinogen level group. This shows fibrinogen could independently predict a high risk of MACCEs in patients with T2DM, even if the SYNTAX score was excluded. Given that the fibrinogen test is relatively simple and inexpensive, it could serve as a biomarker to identify high-risk patients for MACCEs among individuals with T2DM after undergoing PCI.

Research has demonstrated that smoking (49), sedentary (50), and an unhealthy diet (51) can raise fibrinogen levels, while exercise training can decrease levels (52). High-density lipoprotein cholesterol has been shown to have a negative relationship with fibrinogen (53, 54). Furthermore, studies have exhibited that both statins and fibrates can effectively diminish plasma fibrinogen levels, with fibrates appearing to be more successful (55). Previous studies have indicated that fibrinogen levels are higher in diabetic patients than in non-diabetic patients (34), therefore, it is worthwhile to investigate the potential benefits of lowering fibrinogen levels for patients withT2DM.

### 2.4 Limitations

This study has several limitations. Firstly, it is a single-center study with a small sample size, making it challenging to completely eliminate selection bias and confounding factors. To obtain more precise results, it is necessary to increase the sample size and design a prospective multicenter study. Secondly, the majority of patients in this study were only monitored via telephone. As such, the resultant follow-up findings may be influenced by variables such as the working status of the follow-up staff, the degree of patient collaboration, and awareness of the disease. Thirdly, it is important to note that this study focused solely on the baseline fibrinogen levels and did not consider the potential impact of antiplatelet or lipid-lowering medications on fibrinogen levels in the post-PCI period being studied. Finally, our study specifically focused on patients with T2DM who underwent PCI. Therefore, it may not be possible to generalize these findings to the wider population.

### 2.5 Conclusion

Elevated fibrinogen levels were associated with increased coronary anatomical complexity and a higher incidence of MACCEs after PCI in patients with T2DM. Therefore, fibrinogen levels hold potential as a noninvasive biomarker for predicting both coronary anatomical complexity and clinical prognosis in patients with T2DM, facilitating the early identification of individuals at high risk.

## Data availability statement

The raw data supporting the conclusions of this article will be made available by the authors, without undue reservation.

## Author contributions

HS: Writing – original draft. YC: Writing – original draft. QC: Writing – original draft. TY: Writing – original draft. CC: Writing – original draft. XC: Writing – original draft. SY: Writing – original draft. LQ: Writing – original draft. YL: Writing – original draft. SX: Writing – review & editing. LC: Writing – review & editing.
