# Supplementary material for: The association between fibrinogen levels and severity of coronary artery disease and long-term prognosis following percutaneous coronary intervention in patients with type 2 diabetes mellitus
Source: Front Endocrinol (Lausanne). 2023 Nov 29;14:1287855. doi: 10.3389/fendo.2023.1287855 (PMC10716187; doi:10.3389/fendo.2023.1287855)
Supplement: Supplementary file 1 [file DataSheet_1.pdf]

Supplementary Material

1 Supplementary Data

The supplementary Material comprises five tables and a composite figure.

2 Supplementary Figures and Tables

Table S1: Baseline characteristics according to fibrinogen level (Median (IQR))

Variables	Fibrinogen≤3.79g/l, n=312	Fibrinogen>3.79g/l, n=228	P
Male, n (%)	213(68.3)	150(65.8)	0.544
Age, years	69(61,76)	70(62,77)	0.583
Hypertension, n (%)	229(73.4)	172(75.4)	0.592
Previous PCI, n (%)	34(10.9)	20(8.8)	0.416
Previous heart failure, n (%)	15(4.8)	11(4.8)	0.993
COPD, n (%)	7(2.2)	8(3.5)	0.377
Previous stroke, n (%)	12(3.8)	20(8.8)	0.017
peripheral arterial disease, n (%)	3(1.0)	4(1.8)	0.421
Chronic kidney disease, n (%)	5(1.6)	20(8.8)	<0.001
Smoking, n (%)	139(44.6)	105(46.1)	0.972
SBP, mmHg	133(121,148)	133(117.50,147.50)	0.564
DBP, mmHg	76(67.75,84.25)	77(68.00,86.75)	0.474
Heart rate, bpm	76(67,86)	80(71,90)	0.001
Troponin-T, pg/ml	21.00(11.14,177.73)	101.80(21.80,1050.60)	<0.001
BNP, pg/ml	81.10(33.38,202.55)	214.45(66.00,711.10)	<0.001
Creatinine, umol/l	73.70(63.13,89.98)	83.50(67.30,109.10)	<0.001
Uric acid, umol/l	362.45(296.05,433.20)	384.10(302.90,457.40)	0.083
Cystatin c, mg/l	1.13(0.96,1.36)	1.34(1.06,1.74)	<0.001
FBG, mmol/l	7.48(5.93,10.03)	8.25(6.64,11.64)	0.002

Continue Table S1

HbA1c, mmol/l	7.40(6.70,8.60)	7.70(6.70,9.20)	0.129
Triglycerides, mmol/l	1.63(1.14,2.41)	1.57(1.13,2.33)	0.783
Cholesterol, mmol/l	4.13(3.46,4.93)	4.37(3.51,5.24)	0.102
HDL-C, mmol/l	1.10(0.93,1.27)	1.10(0.91,1.31)	0.487
LDL-C, mmol/l	2.50(1.93,3.08)	2.67(2.07,3.28)	0.082
Residual cholesterol, mmol/l	0.54(0.35,0.75)	0.57(0.39,0.81)	0.159
Lipoprotein(a), mg/l	76.80(42.40,192.55)	129.90(72.35,326.30)	<0.001
Apo(A), mmol/l	1.22(1.07,1.38)	1.19(1.00,1.37)	0.061
Apo(B), mmol/l	0.79(0.61,1.02)	0.85(0.68,1.07)	0.024
Homocysteine, umol/l	13.10(10.60,17.60)	13.70(10.43,19.28)	0.373
Albumin, g/l	40.10(37.30,42.70)	38.90(35.25,41.73)	<0.001
AST, IU/l	24.10(17.60,44.43)	25.70(18.30,40.30)	0.647
ALT, IU/l	24.65(17.73,41.63)	25.70(16.70,40.05)	0.756
Direct bilirubin, umol/l	2.68(1.99,3.64)	2.55(1.89,3.80)	0.600
Indirect bilirubin, umol/l	9.78(7.56,12.82)	9.65(7.13,12.73)	0.254
D-dimer, mg/l	0.30(0.18,0.50)	0.52(0.29,0.94)	<0.001
PT, s	13.65(12.50,16.80)	13.85(12.50,16.20)	0.296
INR	0.95(0.91,1.00)	0.95(0.91,1.02)	0.754
APTT, s	37.60(34.50,40.40)	38.10(35.00,41.80)	0.086
RBC count, *10^12/l	4.41(4.00,4.75)	4.26(3.83,4.65)	0.003
Hemoglobin, g/l	135.00(123.00,147.25)	129.00(116.00,143.00)	<0.001
WBC count, *10^9/l	6.66(5.46,8.42)	7.93(6.11,10.03)	<0.001
Platelet count, *10^9/l	155.50(127.50,188.00)	185.00(140.50,223.50)	<0.001
LVEF, %	58(54,62)	56(43,61)	<0.001

Continue Table S1

SYNTAX score	14.50(9.00,20.00)	16(9.63,23.00)	0.017
Residual SYNTAX score	4(0,8)	4(1,9)	0.832
AMI, n (%)	109(34.9)	130(57.0)	<0.001
Diagnosis, n (%)			<0.001
SAP	52(16.7)	27(11.8)	
UA	151(48.4)	71(31.1)	
NSTEMI	48(15.4)	69(30.3)	
STEMI	61(19.6)	61(26.8)	
Angiographic data			
LM, n (%)	14(4.5)	10(4.4)	0.955
MVD, n (%)	222(71.2)	175(76.8)	0.145
Calcified lesions, n (%)	46(14.9)	49(22.3)	0.030
Thrombosis, n (%)	34(11.0)	16(7.3)	0.145
Long lesion, n (%)	75(24.0)	47(20.6)	0.347
CTO, n (%)	58(18.8)	56(25.5)	0.068
Number of stents	1(1,2)	2(1,2)	<0.001
Length of stents, mm	32.00(20.00,52.00)	38.00(23.00,58.25)	0.006
MACCEs, n (%)	52(16.7)	64(28.1)	0.001
All-cause death, n (%)	12(3.8)	23(10.1)	0.004
Stroke, n (%)	9(2.3)	14(6.1)	0.064
Unplanned revascularization, n (%)	31(10.3)	29(13.3)	0.362
Recurrent MI, n (%)	8(2.6)	7(3.1)	0.724
Cardiac death, n (%)	4(1.3)	12(5.3)	0.003

Data are presented as median (IQR) or n (%). BMI: body mass index; PCI: percutaneous coronary intervention; COPD: chronic obstructive pulmonary disease; SBP: systolic blood pressure; DBP: diastolic blood pressure; BNP: B-type natriuretic peptide; FBG: fasting blood glucose; HbA1C: glycosylated hemoglobin A1c; HDL-C: High-Density Lipoprotein Cholesterol, LDL-C: Low-Density Lipoprotein Cholesterol; Apo(A): Apolipoprotein A; Apo(B): Apolipoprotein B; AST: aspartate transaminase; ALT: alanine transaminase; PT: prothrombin time; INR: international normalized ratio; APTT: activated partial thromboplastin time; RBC count: red blood cell count; WBC count: white blood cell count; LVEF: left ventricular ejection fraction; AMI: acute myocardial infarction; SAP: stable angina pectoris, UA: unstable angina; NSTEMI: non-ST-segment elevation myocardial infarction; STEMI: ST-segment elevation myocardial infarction; LM: left main disease; MVD: multivessel disease; CTO: chronic total occlusion; MACCEs: major cardiovascular and cerebrovascular adverse events; MI: myocardial infarction.

Table S2: Baseline characteristics according to fibrinogen level (Median (IQR))

Variables	Fibrinogen≤4.28g/l, n=389	Fibrinogen>4.28g/l, n=151	P
Male, n (%)	260(67.0)	103(68.2)	0.789
Age, years	69(61,76)	68(61,77)	0.859
Hypertension, n (%)	290(74.6)	111(73.5)	0.804
Previous PCI, n (%)	39(10)	15(9.9)	0.975
Previous heart failure, n (%)	16(4.1)	10(6.6)	0.222
COPD, n (%)	11(2.8)	4(2.6)	0.910
Previous stroke, n (%)	18(4.6)	14(9.3)	0.040
Peripheral arterial disease, n (%)	5(1.3)	2(1.3)	0.971
Chronic kidney disease, n (%)	7(1.8)	18(11.9)	<0.001
Smoking, n (%)	176(46.4)	68(45.3)	0.581
SBP, mmHg	133(121,149)	132.5(115,147)	0.235
DBP, mmHg	77(68,85)	76(67.25,85.75)	0.728
Heart rate, bpm	78(68,86)	82(71,94)	0.006
Troponin-T, pg/ml	22.52(11.33,202.65)	182.30(28.66,1365.00)	<0.001
BNP, pg/ml	81(34.00,219.00)	299.30(90.60,875.40)	<0.001
Creatinine, umol/l	73.95(62.9089.98)	89.40(70.68,113.80)	<0.001
Uric acid, umol/l	362.70(301.70,433.70)	393.00(286.68,476.70)	0.099
Cystatin c, mg/l	1.14(0.96,1.36)	1.45(1.13,2.00)	<0.001
FBG, mmol/l	7.61(6.04,10.11)	8.43(6.67,12.56)	0.003
HbA1c, mmol/l	7.40(6.70,8.70)	8.00(6.60,9.30)	0.224
Triglycerides, mmol/l	1.63(1.16,2.41)	1.52(1.12,2.27)	0.264
Cholesterol, mmol/l	4.17(3.46,5.17)	4.39(3.59,5.08)	0.556
HDL-C, mmol/l	1.10(0.93,1.28)	1.06(0.87,1.29)	0.375
LDL-C, mmol/l	2.51(1.95,3.20)	2.63(2.07,3.20)	0.583

Continue Table S2

Residual cholesterol, mmol/l	0.54(0.36,0.79)	0.57(0.39,0.77)	0.623
Lipoprotein(a), mg/l	81.80(44.50,215.70)	132.10(80.70,372.30)	<0.001
Apo(A), mmol/l	1.23(1.07,1.39)	1.12(0.96,1.32)	<0.001
Apo(B), mmol/l	0.80(0.61,1.04)	0.86(0.67,1.03)	0.246
Homocysteine, umol/l	13.00(10.43,17.70)	14.10(11.20,19.90)	0.096
Albumin, g/l	40.20(37.30,42.70)	38.00(34.50,40.85)	<0.001
AST, IU/l	24.30(17.90,43.80)	26.30(18.30,41.25)	0.611
ALT, IU/l	25.10(17.70,40.70)	26.20(16.00,40.75)	0.911
Direct bilirubin, umol/l	2.59(1.97,3.54)	2.68(1.94,4.02)	0.707
Indirect bilirubin, umol/l	9.77(7.47,12.72)	9.73(6.70,12.84)	0.341
D-dimer, mg/l	0.36(0.25,0.64)	0.64(0.38,1.01)	<0.001
PT, s	13.40(12.50,16.70)	14.20(12.70,16.20)	0.434
INR	0.94(0.91,1.00)	0.96(0.92,1.05)	0.009
APTT, s	37.10(34.40,40.13)	39.15(35.58,43.33)	<0.001
RBC count, *10^12/l	4.42(4.00,4.77)	4.18(3.71,4.53)	<0.001
Hemoglobin, g/l	135.00(123.00,147.25)	126.00(110.00,139.00)	<0.001
WBC count, *10^9/l	6.74(5.48,8.60)	8.19(6.35,10.15)	<0.001
Platelet count, *10^9/l	158.50(129.00,191.25)	187.00(142.50,232.50)	<0.001
LVEF, %	58(53,62)	55(42,60)	<0.001
SYNTAX score	14(8.13,20.00)	16(10,22.50)	0.004
Residual SYNTAX score	4(0,8)	4(1,9)	0.620
AMI, n%	143(36.8)	96(63.6)	<0.001
Diagnosis, n%			<0.001
SAP	66(17.0)	13(8.6)	
UA	180(46.3)	42(27.8)	

Continue Table S2

NSTEMI	67(17.2)	50(33.1)	
STEMI	76(19.5)	46(30.5)	
Angiographic data			
LM, n (%)	18(4.6)	6(4.0)	0.741
MVD, n (%)	277(71.2)	120(79.5)	0.051
Calcified lesions, n (%)	65(17.0)	30(20.7)	0.321
Thrombosis, n (%)	40(10.4)	10(6.9)	0.214
Long lesion, n (%)	93(23.9)	29(19.2)	0.241
CTO, n (%)	77(20.1)	37(25.5)	0.177
Number of stents	1(1,2)	2(1,2)	0.018
Length of stents, mm	33.00(20.00,52.50)	38.00(23.0058.25)	0.044
MACCEs, n (%)	67(17.2)	49(32.5)	<0.001
All-cause death, n (%)	16(4.1)	19(12.6)	<0.001
Stroke, n (%)	12(3.1)	11(7.3)	0.030
Unplanned revascularization, n (%)	40(10.3)	20(13.3)	0.318
Recurrent MI, n (%)	8(2.1)	7(4.6)	0.102
Cardiac death, n (%)	6(1.5)	10(6.6)	0.002

Data are presented as median (IQR) or n (%). Abbreviations as shown in Table S1.

Table S3: Logistic regression analysis within the subgroup

Variables	N	Unadjusted OR(95%CI)	p	Adjusted OR[a] (95%CI)	P
SAP	79	1.053(0.662-1.676)	0.826	1.352(0.681-2.686)	0.389
ACS	416	1.170(1.014-1.351)	0.027	1.195(1.008-1.402)	0.031
UA	222	1.393(1.004-1.791)	0.033	1.164(1.021-1.473)	0.042
MI	239	1.064(0.904-1.254)	0.454	0.822(0.589-1.148)	0.251

[a]Adjusted covariates includes Chronic kidney disease(CKD), Heart rate, Troponin-T, B-type natriuretic peptide (BNP), Cystatin c, Residual cholesterol, Apolipoprotein B (Apo(B)), Albumin, Direct bilirubin, Indirect bilirubin, Fibrinogen, D-dimer, RBC count, Hemoglobin, left ventricular ejection fraction (LVEF). OR, Odds ratio; CI, confidence interval; N, number of patients. Other abbreviations are as in Table S1.

Table S4: COX regression analysis within the subgroup

Variables	N	Unadjusted HR(95%CI)	p	Adjusted HR[b](95%CI)	P
**SAP**	79				
MACCEs	15	1.052(0.651-1.699)	0.836	1.040(0.422-2.560)	0.932
All-cause death	5	1.289(0.866-1.919)	0.212	2.245(0.640-7.876)	0.207
Stroke	3	0.923(0.278-3.063)	0.896	0.839(0.149-4.723)	0.843
Unplanned revascularization	8	0.538(0.173-1.669)	0.283	0.001(0.000-42.38)	0.205-
Recurrent MI	0	-	-	-	-
Cardiac death	0	-	-	-	-
**ACS**	461				
MACCEs	101	1.148(1.029-1.280)	0.013	1.223(1.021-1.444)	0.029
All-cause death	30	1.170(0.951-1.438)	0.137	1.06(0.823-1.366)	0.651
Stroke, n (%)	20	1.163(0.920-1.471)	0.206	1.146(0.862-1.523)	0.349
Unplanned revascularization	53	1.118(0.954-1.311)	0.168	1.121(0.897-1.402)	0.315
Recurrent MI	15	1.132(0.839-1.526)	0.418	1.030(0.645-1.645)	0.902
Cardiac death	15	1.210(0.950-1.539)	0.122	1.121(0.803-1.566)	0.502
**UA**	222				
MACCEs	48	1.616(1.341-1.949)	<0.001	1.638(1.318-2.036)	<0.001
All-cause death	12	1.679(1.223-2.303)	0.001	1.493(1.032-2.160)	0.033
Stroke	13	1.716(1.134-2.596)	0.011	1.732(0.959-3.129)	0.069
Unplanned revascularization	24	1.581(1.192-2.098)	0.001	1.692(1.226-2.334)	0.001
Recurrent MI	5	0.566(0.137-2.335)	0.431	0.123(0.007-2.104)	0.148
Cardiac death	7	1.608(1.121-2.307)	0.010	1.317(0.876-1.981)	0.186

Continue Table S4

**MI**	239				
MACCEs	53	1.059(0.906-1.237)	0.471	0.991(0.804-1.220)	0.931
All-cause death	18	1.038(0.726-1.413)	0.814	0.816(0.528-1.261)	0.360
Stroke	7	1.148(0.769-1.713)	0.500	1.150(0.717-1.844)	0.563
Unplanned revascularization	29	1.001(0.800-1.253)	0.991	0.856(0.588-1.245)	0.415
Recurrent MI	10	1.096(0.785-1.531)	0.589	0.967(0.514-1.819)	0.918
Cardiac death	8	1.100(0.769-1.574)	0.602	1.013(0.603-1.700)	0.961

[b]Adjusted covariates includes Chronic kidney disease(CKD), Age, Sex, Heart rate, Creatinine, Uric acid, Cystatin c, Lipoprotein(a), Homocysteine, aspartate transaminase(AST), alanine transaminase(ALT), Fibrinogen, D-dimer, left ventricular ejection fraction (LVEF), Syntax score, multivessel disease(MVD), Calcified lesions, chronic total occlusion(CTO). HR, hazard ratio; CI, confidence interval; N, number of patients. Other abbreviations are as in Table S1.

Table S5: The AUROC of fibrinogen predicted the mid/high SYNTAX score and clinical endpoints.

Variables	endpoint	AUROC (95%CI)	P	endpoint	AUROC (95%CI)	P
**SAP**	Mid/high SYNTAX score	0.572(0.405-0.739)	0.465			
	MACCEs	0.497(0.314-0.679)	0.970	All-cause death	0.668(0.395-0.940)	0.212
	Stroke	0.566(0.294-0.837)	0.700	Cardiac death	0.923(0.864-0.982)	0.148
	Recurrent MI	-	-	Unplanned revascularization	0.382(0.149-0.615)	0.276
**ACS**	Mid/high SYNTAX score	0.609(0.549-0.670)	0.001			
	MACCEs	0.609(0.551-0.667)	<0.001	All-cause death	0.647(0.546-0.749)	0.004
	Stroke	0.612(0.498-0.727)	0.068	Cardiac death	0.687(0.547-0.826)	0.011
	Recurrent MI	0.572(0.427-0.718)	0.340	Unplanned revascularization	0.552(0.481-0.623)	0.186
**UA**	Mid/high SYNTAX score	0.607(0.532-0.682)	0.040			
	MACCEs	0.686(0.604-0.768)	<0.001	All-cause death	0.751(0.617-0.885)	0.003
	Stroke	0.700(0.574-0.826)	0.016	Cardiac death	0.810(0.667-0.953)	0.005
	Recurrent MI	0.417(0.315-0.518)	0.524	Unplanned revascularization	0.591(0.476-0.705)	0.146

Continue Table S5

**MI**	Mid/high SYNTAX score	0.567(0.490-0.643)	0.112			
	MACCEs	0.581(0.494-0.668)	0.072	All-cause death	0.577(0.425-0.730)	0.276
	Stroke	0.612(0.350-0.874)	0.314	Cardiac death	0.574(0.338-0.809)	0.478
	Recurrent MI	0.600(0.434-0.766)	0.284	Unplanned revascularization	0.542(0.449-0.634)	0.467

AUROC, the area under the receiver operating characteristic curve; CI, confidence interval; Other abbreviations are as in Table S1.

2.1 Supplementary Figures

Figure F1: Kaplan-Meier survival analysis of the clinical endpoint.

Figure legend: (A): Kaplan-Meier survival analysis of all-cause death (log-rank: P < 0.01). (B): Kaplan-Meier survival analysis of cardiac death (log-rank: P < 0.01). (C): Kaplan-Meier survival analysis of stroke (log-rank: P < 0.05). (D): Kaplan-Meier survival analysis of unplanned revascularization (log-rank: P > 0.05). (E): Kaplan-Meier survival analysis of recurrent myocardial infarction (log-rank: P > 0.05). MACCEs major adverse cardiovascular and cerebrovascular events.

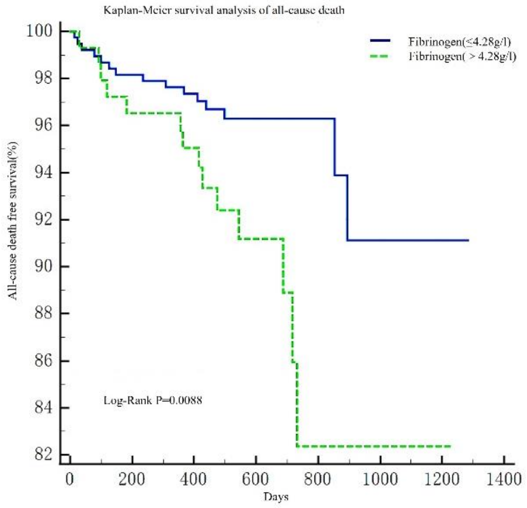

(A)

(A): Kaplan-Meier survival analysis of all-cause death (log-rank: P < 0.01).

(B): Kaplan-Meier survival analysis of cardiac death (log-rank: P < 0.01).

(C): Kaplan-Meier survival analysis of stroke (log-rank: P < 0.05).

(D): Kaplan-Meier survival analysis of unplanned revascularization (log-rank: P > 0.05).

(E): Kaplan-Meier survival analysis of recurrent myocardial infarction (log-rank: P > 0.05).

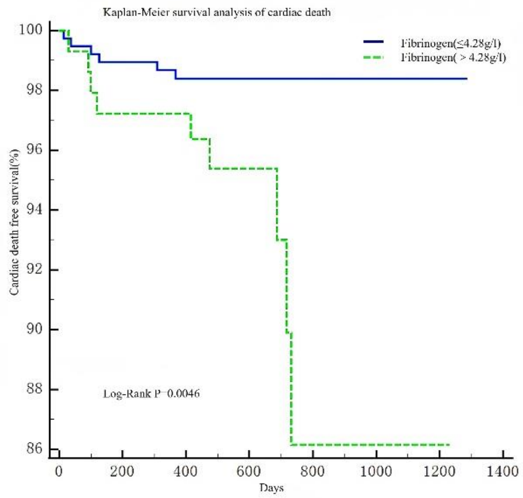

(B)

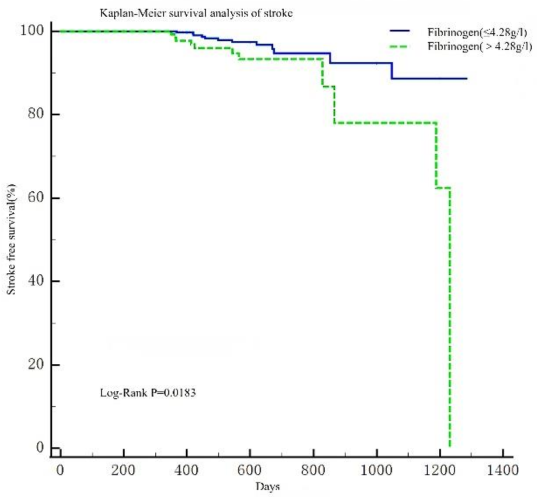

(C)

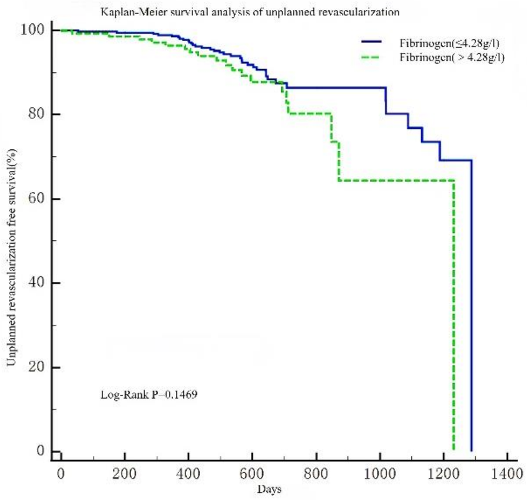

(D)

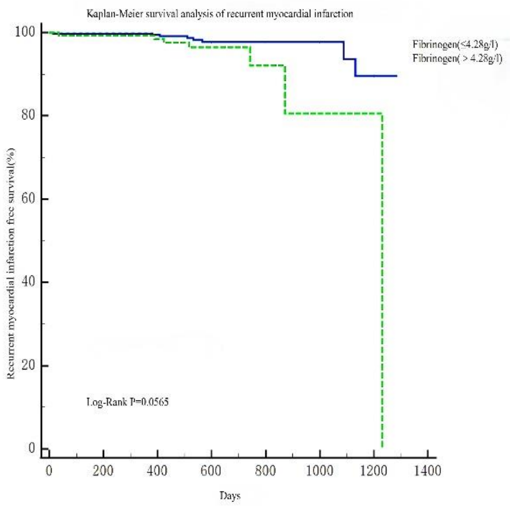

(E)
